# Topographic contrast of ultrathin cryo-sections for correlative super-resolution light and electron microscopy

**DOI:** 10.1038/srep34062

**Published:** 2016-09-26

**Authors:** José María Mateos, Bruno Guhl, Jana Doehner, Gery Barmettler, Andres Kaech, Urs Ziegler

**Affiliations:** 1 Center for Microscopy and Image Analysis, University of Zurich, Switzerland

## Abstract

Fluorescence microscopy reveals molecular expression at nanometer resolution but lacks ultrastructural context information. This deficit often hinders a clear interpretation of results. Electron microscopy provides this contextual subcellular detail, but protein identification can often be problematic. Correlative light and electron microscopy produces complimentary information that expands our knowledge of protein expression in cells and tissue. Inherent methodological difficulties are however encountered when combining these two very different microscopy technologies. We present a quick, simple and reproducible method for protein localization by conventional and super-resolution light microscopy combined with platinum shadowing and scanning electron microscopy to obtain topographic contrast from the surface of ultrathin cryo-sections. We demonstrate protein distribution at nuclear pores and at mitochondrial and plasma membranes in the extended topographical landscape of tissue.

The function of a specific protein can be better understood once its subcellular localization within a tissue is known. Super-resolution light microscopy can image fluorescence molecules with a spatial resolution between 10–90 nm^1^,^2^. The non-fluorescent cellular context in which proteins are located is however invisible with these methods. Co-localization with other fluorescence markers may partially overcome this limitation and obtain some reference points in the complexity of the tissue.

On the other hand, electron microscopy (EM) provides excellent ultrastructural information with accurate subcellular localization to <2 nm, but EM immunostaining experiments with several markers simultaneously are difficult, and can be demanding to execute. In addition, finding rare events or the analysis of protein expression in a large population of cells requires more exhaustive and time-consuming quantifications.

Correlative light and electron microscopy methods (CLEM) must incorporate additional preparative techniques to correctly accommodate the differences between the two methods to reap the combined advantages of both techniques^3^. EM methods for protein localization traditionally require ultrathin sections from samples that are solidified either by polymerization in resins or by freezing protocols. From the first group, CLEM sample preparation methods have recently been improved to preserve fluorescence molecules after resin embedding^4^, and maintain accessibility to intracellular epitopes^5^. Sample extraction and shrinkage caused by dehydration still limits ultrastructural preservation of the sample. This can be greatly improved by high-pressure freezing, freeze substitution and embedding of the samples in hydrophilic resins^6^,^7^,^8^. The second group of methods obtains ultrathin sections based on solidifying the sample by freezing. Ultrathin cryo-sections, *e.g.*, Tokuyasu sections^9^ preserve tissue ultrastructure and antigenicity and facilitate CLEM of hydrated samples^10^,^11^,^12^,^13^,^14^,^15^. Tokuyasu sections can exhibit weak or negative contrast obtained after post-staining with heavy metals^16^ rendering interpretation of data difficult for some experiments. Recent developments in contrast enhancement for Tokuyasu sections are facilitating structural sample identification^17^. Alternatively, cryo-sections prepared for the platinum replica technique^18^ has been used to correlate stochastic optical reconstruction microscopy (STORM) and transmission electron microscopy^19^. However, the replica method still involves complex transfer steps^19^. Our new approach introduces two new main steps, namely, collecting Tokuyasu sections on silicon wafers and applying contrast with platinum by electron beam evaporation, providing a quick, simple and consistent way to obtain correlative image information from tissue samples.

## Results

### Collection of cryo-sections on silicon wafers facilitates CLEM

**Figure 1:**
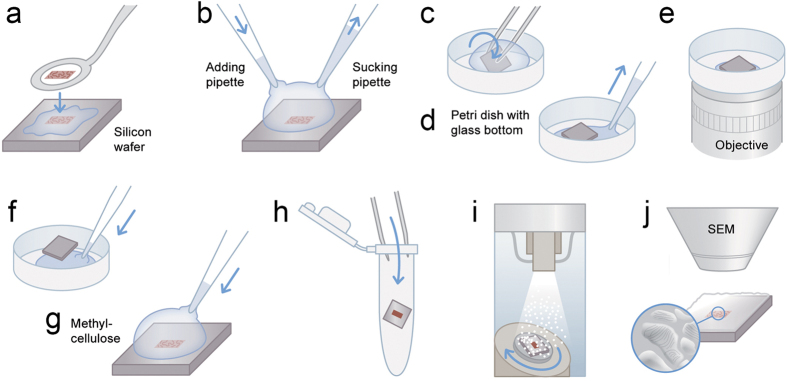
Scheme of CLEM method. (**a**) 100 nm ultrathin-cryosections are transferred from the cryo-ultramicrotome to the surface of a silicon wafer. This step is performed with a perfect loop and a drop of sucrose and methylcellulose. (**b**) Two pipettes are used to exchange solutions for immunolabelling so that sections remain wet. (**c**) The wafer is transferred upside down to a petri dish (glass bottom) with a drop of a 1:1 mixture of glycerin and PBS or imaging buffer for super-resolution, respectively. (**d**) A pipette is used to remove excess liquid from the sides of the wafer to bring the wafer nearly to the bottom of the petri dish (a very thin layer of solution is maintained). (**e**) Light microscopy is performed on inverted microscopes. (**f**) After imaging, a drop of PBS is applied from the side that allows the liquid to enter the space between wafer and the bottom. This gentle step will bring the wafer to the top of the drop and allows the wafer to be picked up without damaging or losing the section. (**g**) A drop of methylcellulose is added at 4 °C for 1 minute. (**h**) The wafer is transferred with forceps into an Eppendorf tube and immediately centrifuged at 14100×g for 90 sec. (**i**) The specimen is coated with Pt/C by unidirectional or rotary shadowing at an angle of 8°. (**j**) Imaging by SEM using secondary electrons. Design work produced by T. Gschwend, Multimedia and E-Learning Services, University of Zurich.

**Figure 2:**
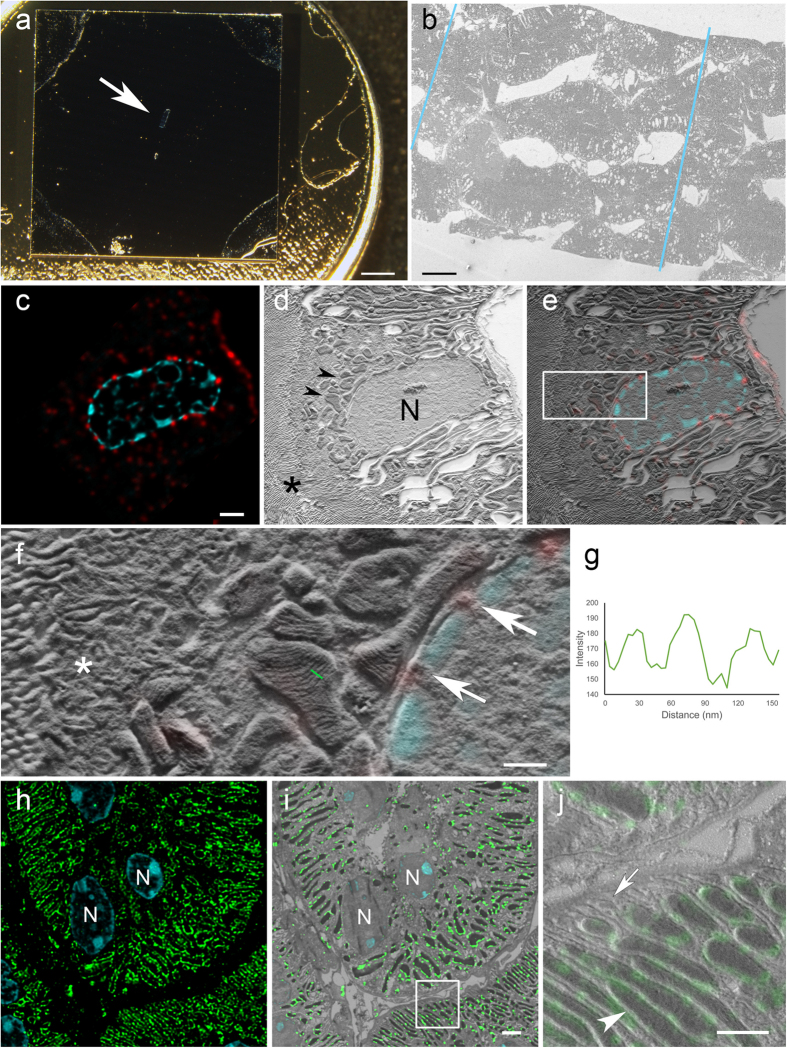
CLEM on silicon wafers. (**a**) Tokuyasu section collected on a silicon wafer and mounted on a SEM stub. Arrow points to the section in the middle of the wafer. (**b**) Serial kidney Tokuyasu sections imaged at low magnification in an SEM. The borders of the sections on the image are highlighted with blue lines. (**c**) De-convolved confocal laser scanning microscopy image of a kidney Tokuyasu section stained with anti-nuclear pore complex protein (red), and DAPI for nuclei (cyan). The ultrathin section (100 nm) shows the distribution of the nuclear pores along the nuclear membrane. (**d**) SEM image of the same area. The topographical information from the tissue reveals the structure of mitochondria (arrowheads), nuclei (N) and microvilli (asterisk). (**e**) Overlay of (c–d) (f) High magnification image of white box in e. Asterisk labels a vesicular area, arrows point to nuclear pores identified by the correlation and in the middle of the image a group of mitochondria can be observed. Mitochondrial cristae are resolved. Images d and f acquired with Inlens SE detector. Specimen shadowed unidirectionally with 2 nm Pt/C at 8° (**g**) Line plot (with inverted grey values) along the cristae in f (green line) shows the topography of the cristae with an inter cristae distance of around 30 nm. (**h**) Overview of a confocal laser scanning microscopy image showing the expression of Tom20 (green) on mitochondrial outer membranes. The thin sections reveal “sharp”, round or elongated shapes that corresponds to the morphology of the corresponding mitochondria. DAPI staining (cyan) was used for the alignment of the images. Nuclei (N). (**j**) White box in (i), the tissue landscape clearly shows the differentiation of mitochondria (arrowhead) and basolateral infoldings (arrow). Image i acquired with InLens SE detector, image j with SE2 detector. Specimen rotary shadowed with 2 nm Pt/C at 8°. Scales: (**a**) 2 mm; (**b**) 20 μm; (**c**) 2 μm; (**f**) 0.5 μm; (**i**) 2 μm; (**j**) 1 μm.

100 nm-thick Tokuyasu ultrathin kidney cryo-sections were collected on a 7 × 7 mm silicon wafer (Figs 1a and 2a). The flat silicon surface provides stability, facilitates the handling of the sections compared to EM grids, reduces the deformations in the tissue caused by drying or exposure to a vacuum, and minimizes folds and breaks^20^ that are often found in sections collected on formvar film-coated grids^21^ (Fig. 2b). This conductive support prevents charging artefacts and is therefore ideal for scanning electron microscopy (SEM) imaging^21^. Sections were subsequently processed for immunofluorescence staining (Fig. 1b). For light microscopy (LM), a thin layer of buffer and glycerin (1:1) was retained between the wafer and the glass-bottom petri dish. This mixture keeps the sections wet and close to the coverslip (Fig. 1c–e). Optical imaging was performed with confocal laser scanning microscopes using a closed pinhole to Airy unit 0.7 followed by deconvolution (Supplementary Fig. 1; Huygens, SVI, Netherlands). After confocal microscopy, a short postfixation with glutaraldehyde was followed by embedding in methylcellulose and centrifugation of the wafer to cover the ultrathin Tokuyasu section with a thin layer of dried methylcellulose (Fig. 1f–h). This latter step protects the tissue from drying artefacts^16^ and avoids the need of using solvents and embedding in resin, which contribute to major problems in tissue extraction and shrinkage. Nevertheless, shrinkage occurs to some extent during drying in methylcellulose^13^. The sections were platinum/carbon shadowed to generate topographic contrast for SEM by detecting secondary electrons (Fig. 1i,j).

The fluorescent nuclear stain DAPI (4′,6-diamidino-2-phenylindole dihydrochloride) was used as a first rough reference to correlate the confocal and SEM images (Fig. 2c–e). The nuclear pore complex protein was observed along the nuclear membrane in the confocal and in the scanning electron microscopy image in relation to the different cellular components (Fig. 2f). Mitochondria, vesicles and microvilli exhibit high topographic contrast. Mitochondrial cristae are clearly visible, demonstrating the level of resolution this novel technique can provide (Fig. 2f,g).

All SEM images were taken using either an Inlens SE (SE I) or side mounted SE II detector. Contrast changes, in particular in mitochondria, are much more prominent when using the Inlens SE detector. Areas between dense cristae do not contain platinum or only tiny amounts (due to low angle coating of 8°) and only few secondary electrons escape from this area providing a “dark” area. On the membranes of the cristae itself (sticking out), platinum is added in larger amounts by the low angle shadowing and produce a high contrast due to more platinum and etch effects. The reduced resolution with the SE II detector (virtually larger scanning spot) also leads to an averaging of the signal and therefore less contrast between membranes of cristae and areas between cristae, “washing out” high contrast differences in the nm scale.

### Conventional and super-resolution microscopy combined with SEM

**Figure 3:**
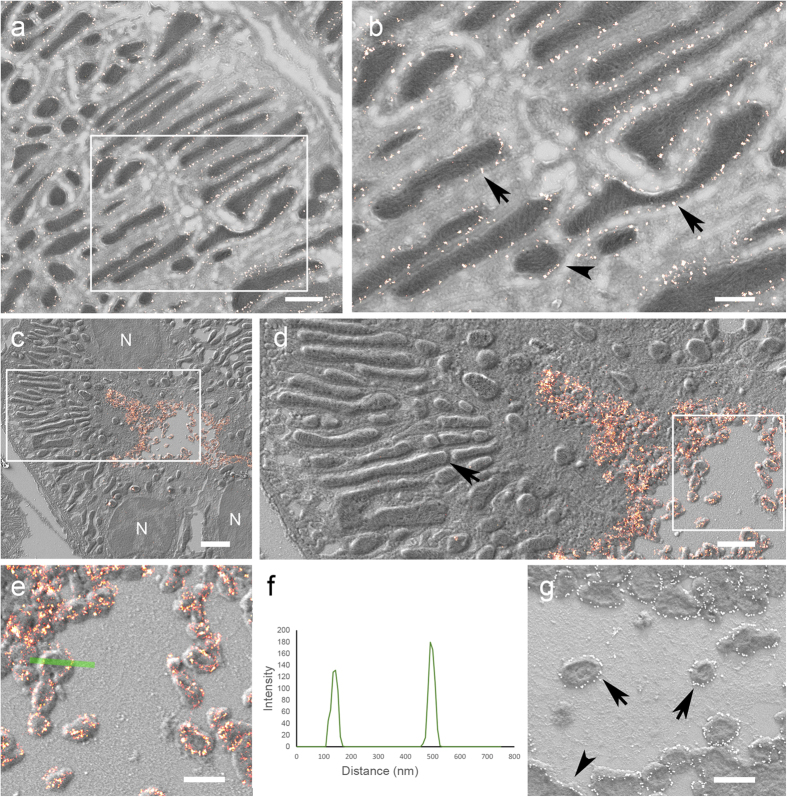
Correlative super-resolution LM and SEM (**a**) Tom20 expression (bright red points) by GSDIM and correlation with SEM. (**b**) White box in a. Elongated (arrows) and round (arrowhead) cross-sections of mitochondria are decorated at the external membrane with Tom20 staining shown with super-resolution microscopy. Images (a) and (b) acquired with InLens SE detector, specimen rotary shadowed with 2 nm Pt/C at 8°. (**c)** Overview image of super-resolved localization of pNCC (bright red signal) at the luminal plasma membrane of a distal tubulus. Nuclei (N). (**d**) High magnification of white box in c showing the precise correlation of the fluorescent and the electron micrographs. pNCC is localized at the plasma membrane and in microvilli. Mitochondrial cristae are clearly visible (arrow). (**e**) High magnification of white box in d showing GSDIM and SEM image. Some transversally cut apical microvilli show the labelling at the membranes. (**f**) Line plot from green line in e showing the profile of the sectioned microvilli and labelling precision. (**g**) Immunogold labelling produces a similar pattern of protein expression. Immunogold particles (white dots) accumulate along the plasma membrane (arrowhead) and transversally cut apical microvilli (arrows) appear surrounded by gold particles. Images (a; b; g acquired with In lens SE detectors. (c–e) acquired with SE2 detector. Specimens were rotary shadowed with 2 nm Pt/C 8° in images (a–e) and unidirectionally in image (g) Scale bars: (**a**) 1 μm; (**b**) 0.5 μm; (**c**) 2 μm; (**d**) 1 μm; (**e,g**) 0.5 μm.

The above methodology was used to determine the localization of the protein Tom20, a subunit of the translocase mitochondrial outer membrane complex^22^ with confocal microscopy and SEM (Fig. 2h,j) and by super-resolution microscopy (ground state depletion microscopy followed by individual molecule return, GSDIM) and SEM (Fig. 3a,b). The confocal images exhibit elongated or ring-like structures which, when overlaid onto the matching SEM image, correspond to the outer membrane of the mitochondria in these thin sections (Fig. 2j). A similar correlation was possible using super-resolution microscopy (Fig. 3a,b). GSDIM images however exhibited a sparser signal along the outer membrane of the mitochondria. This appears a more accurate immunolabelling pattern since it corresponds with the reported cluster expression of this protein^23^. Finally, we located the phosphorylated form of the NaCl cotransporter (pNCC) at the apical plasma membrane of distal tubules^24^ by super-resolution microscopy. Transversally cut microvilli were identified by super-resolution microscopy. Images display strong staining along the membrane below the diffraction limit of resolution (Fig. 3c–f). The direct immunogold method on Tokuyasu sections (Fig. 3g) was applied to compare the level of accuracy between these two approaches. Both methods showed similar structural resolution and labelling patterns. The immunogold method on silicon wafers could be used to obtain protein localization information on large areas. However, super-resolution microscopy with SEM allows a quicker overview signal detection and identification of rare events (Fig. 3c). Additionally, this method can be applied to multiple labelling approaches and in other tissues and cells (Supplementary Figs 2 and 3).

## Discussion

We provide a relatively quick, facile and reliable method for CLEM in cells and tissue. A number of correlative approaches are currently available to detect endogenous proteins^3^,^25^. Among those methods some identify proteins at subcellular level in tissue overcoming the challenges of 3D complexity, sample preparation, preservation of antigenicity and structure^12^,^26^,^19^. Tokuyasu sections from cells and tissues are hydrated, and remain so during the labelling procedure; this preserves the ultrastructure and antigenicity of most epitopes. Immunofluorescence and immunogold labelling are routinely used techniques^27^ that allow imaging of wet samples at super-resolution followed by transfer to electron microscopes without resin embedding^10^ and associated potential artefacts^28^. Tokuyasu sections treated with standard contrast agents often appear poor in contrast. Recent procedures however, have introduced steps to increase sample contrast^17^. In this study we propose a simple solution to this problem; the application of a thin (2 nm) layer of platinum/carbon on the tissue surface as an effective method for contrast generation without the need for heavy metal post-staining. This contrast corresponds to the roughness or topography of the tissue section^29^,^30^,^31^. In addition, the platinum/carbon layer facilitates image acquisition by reducing possible charging artefacts during imaging of biological specimens. We demonstrate that topographic contrast is able to precisely resolve mitochondrial cristae, vesicles, and microvilli in cells of kidney tissue.

The excellent intrinsic axial resolution (100 nm section thickness) of the Tokuyasu sections provides better resolution than optical sectioning of thick samples^32^. In addition, we have imaged these 100 nm thin sections with confocal laser scanning microscopes using a closed pinhole [Airy unit 0.7] followed by deconvolution (Huygens, SVI, Netherlands). This generates higher resolution images in the XY plane than those acquired with widefield microscopes^33^. This combination provides exceptional XYZ resolution for accurate protein localization.

Collecting thin sections on silicon wafers offers many advantages^21^, among them stability, flatness and ease in sample manipulation throughout the processing procedure. Image acquisition by light and electron microscopy is much more stable on silicon than imaging sections on electron microscopy grids^21^. Handling of wafers for incubation with antibodies is also easier and facilitates transfer between instruments without damaging sections. Treated glass coverslips are also used for CLEM methods^34^,^35^. The main advantage of silicon wafers compared with glass is that they are highly conductive and very clean without the need of any pre-treatment. We have developed this method so that once Tokuyasu sections are collected on the wafer, the procedure can be completed in one day with simple steps. We also took into account the equipment commonly accessible to many research groups and the use of open-source software^36^ for the alignment of correlated images.

The complete substitution of standard heavy metal contrast agents by platinum deposition brings homogeneous contrast and stability to the sample. However, fine structures with reduced topographic details are difficult to resolve (ER, microtubules) and complex homogeneous regions (neuropil in brain tissue) are difficult to identify (Supplementary Fig. 3). Platinum replica protocols for cells^37^ and tissue^19^ have been applied to obtain correlative super-resolution microscopy with 3D electron microscopy information. Further developments of the protocol could provide adequate conditions for the finest structural elements. Another limitation of the present technique is the application to serial sections^26^, obtaining serial Tokuyasu sections are challenging and often only with few sections possible.

Our approach incorporates the Tokuyasu technique with simple and quick steps to perform routine correlative imaging at subcellular resolution using standard equipment. The direct topographic contrast obtained by this method represents a novel approach; visualizing sections without need of heavy metal solutions or replica preparation.

## Material and Methods

All animal experiments were conducted according to Swiss Laws and approved by the veterinary administration of the Canton of Zurich, Switzerland. Adult mice (n = 3) were anaesthetized with isoflurane (Baxter) inhalation combined with intracutaneous Temgesic (Reckitt Benckiser) followed by perfusion with 4% formaldehyde in cacodylate buffer (0.1 M, pH 7.4). Kidneys were transversally sectioned to 500 micron thick using a vibratome (Pelco 101, Vibratome R Series 1000, Ted Pella, Inc.) and postfixed with 2% formaldehyde and 0.025% glutaraldehyde in cacodylate buffer for 16 hours at 4 °C. These sections were then cut with a scalpel into 1 mm long pieces and immersed in 2.3 M sucrose overnight at 4 °C and stored at −20 °C.

### Cryosectioning

Pieces of kidney cortex were mounted on “cryo-pins” (Baltic Präparation, Germany #16701950), frozen in liquid nitrogen and transferred to a cryo-ultramicrotome (Ultracut EM FC6, Leica Microsystems). 100 nm thin sections cut with a cryo-immuno diamond knife (Diatome) were collected with a “Perfect loop” (Diatome) with 2% methylcellulose (M-6385; Sigma) and 2.3 M sucrose (1:1)^27^ and transferred to a 7 × 7 mm silicon wafer (Si-Mat Silicon Materials). Sections were stored at 4 °C until further processing.

### Immunolabelling

Sections on the wafers were washed with PBS (0.1 M, pH 7.4, 4 °C) for 20 min followed by incubation with 0.15% glycine in PBS (3 × 1 min) and washes in PBS (3 × 10 sec). Blocking solution composed of 0.5% BSA (Albumin Fraction V, Applichem) and 0.2% gelatin (from bovine skin, Sigma) in PBS was applied for 5 min. Primary antibodies, anti-NuP complex, (4 μg/ml, mAB414, Covance), anti-Tom20 (1 μg/ml, FL-145, Santa Cruz) and pNCC (1:250, antibody kindly provided by Prof. Johannes Loffing, University of Zurich, Switzerland) dissolved in blocking solution were incubated for 40 min at room temperature. After washes with blocking solution (6 × 20 sec) wafers were incubated with secondary antibodies (anti-mouse Alexa Fluor 568, 8 μg/ml, Life Technologies; anti-rabbit Alexa Fluor 488, 8 μg/ml, Life Technologies; anti-rabbit Alexa Fluor 647 F(ab’)_2_, 6 μg/ml Jackson Immuno-Research for GSDIM experiments) in blocking solution (40 min at room temperature). For the immunogold experiments, 12 nm gold conjugated goat anti-rabbit antibodies were incubated for 40 min at room temperature (Optical density OD_525_ 0.15). Washes in blocking solution (6 × 2 min) were followed by PBS (3 × 2 min) and post-fixation with 0.025% glutaraldehyde in PBS for 5 min. Finally, the wafers were transferred to a 6 well plate filled with PBS. Sections were stored at 4 °C until further processing.

### Light microscopy imaging

Before imaging, DAPI (4′,6-diamidino-2-phenylindole dihydrochloride, Sigma, 1:250 dilution) was applied for 10 sec followed by washes in PBS (3 × 10 sec). To avoid drying of the sample, the sections were incubated in a solution of glycerin (80%) and PBS (1:1) for 10 sec and then transferred with the section facing down to a glass bottom (thickness 170 μm +/−5 μm) petri dish (Ibidi). A pipette was used to remove most of the liquid underneath the wafer in order to bring it as close as possible to the bottom of the petri dish.

Confocal laser scanning microscopy was performed on Leica SP5 and SP8 inverted microscopes (Leica Microsystems) with a 63x/1.4 NA oil immersion objective. Image stacks were acquired (60 × 60 × 170 nm) with a pinhole closed to 0.7 Airy units.

DAPI (405 nm excitation) signals were acquired using a PMT detector, Alexa 488 (488 nm excitation) and Alexa 568 (561 nm excitation) signals were acquired with HyD detectors (Leica Microsystem). Deconvolution was accomplished with Huygens Professional (Scientific Volume Imaging B.V.).

### Super-resolution imaging

Wafers were mounted section side down in a petri dish (glass bottom, thickness 170 μm +/−5 μm, Ibidi) onto a drop of a 1:1 mixture of glycerin (80%) and an imaging buffer containing an oxygen scavenging system (200 mM Phosphate buffer containing 10% glucose, 0.5 mg/ml glucoseoxidase, 40 μg/ml catalase, 15 mM beta-Mercaptoethylamine (MEA), pH 8.0). Excess buffer was removed until the wafer was brought as close as possible to the cover glass.

Super-resolution imaging was performed on a Leica SR-GSD 3D microscope (Leica Microsystems) using a 160x (NA 1.43) oil immersion objective (HC PL APO, Leica Microsystems). The system was equipped with 488 nm/300 mW, 532 nm/500 mW and 647 nm/500 mW continuous wave lasers and an EMCCD camera (iXon Ultra, DU-897U, Andor).

The sample was first illuminated with the 642 nm laser at maximum laser power to ensure an efficient transfer of the fluorescent molecules into the off-state. Images of 180 × 180 pixels were then taken with an integration time of 15 ms in epifluorescence mode. A total of 30,000 frames were collected for each reconstruction.

Image reconstruction and visualization via Gaussian fitting was performed with the LAS AF software (Leica Microsystems).

### Shadowing

Once LM imaging was performed the wafers were “lifted” up by application of buffer solution close to the edges of the wafer. After few washes in PBS (3 × 10 min) the wafers were quickly washed with 2% methylcellulose (Sigma-Aldrich) (2 × 5 sec) and left in a drop of methylcellulose at 4 °C for 1–2 min. The wafer was then placed, as fast as possible, in an Eppendorf tube and centrifuged at 14100 × g for 90 sec, mounted on a SEM aluminum stub (Agar Scientific) using an adhesive carbon pad, left drying at room temperature for 5 min and transferred to an electron beam evaporator (MED 020, Leica Microsystems). The specimen was then coated with Platinum/carbon (Pt/C, 2 nm) by unidirectional or rotary shadowing at an angle of 8 degrees (Supplementary Fig. 4).

### Scanning electron microscopy imaging

Sections were imaged with a Zeiss Supra 50 VP and Zeiss Auriga 40 SEM (large images above 4096 × 4096 pixels were acquired with the FIBICS Nanopatterning and Visualization Engine, FIBICS Incorporated) at an acceleration voltage of 1.5 keV, with a 30 μm aperture and an InLens SE detector at a working distance of 2 mm or an SE2 detector at 5 mm working distance. Images were acquired at 4–5 nm pixel size.

### Registration of LM and EM images

Alignment of light and electron microscopy images was done with TrakEM2^36^ within the open-source platform Fiji^38^. Firstly, the LM image was aligned based on manually inserted landmarks from the nuclear DAPI signal and their corresponding signals on the SEM image. After this rough alignment, a finer alignment was performed by registering the centre of several (3 to 5) clearly identified labelled structures (example, round mitochondria expressing Tom20) and their corresponding signals on the SEM image. We used the elastic alignment algorithm in TrakEM2^36^. For data presentation, the light and electron microscopy images were merged with the merge channels plug-in from the open source image processing software Fiji.

## Additional Information

**How to cite this article**: Mateos, J. M. *et al*. Topographic contrast of ultrathin cryo-sections for correlative super-resolution light and electron microscopy. *Sci. Rep.* **6**, 34062; doi: 10.1038/srep34062 (2016).

## Supplementary Material

Supplementary Information

## References

[b1] Li D. . Extended-resolution structured illumination imaging of endocytic and cytoskeletal dynamics. Science (80-.). 349, aab3500–aab3500 (2015). 10.1126/science.aab3500 PMC4659358 26315442

[b2] Betzig E. . Imaging intracellular fluorescent proteins at nanometer resolution. Science 313, 1642–1645 (2006). 16902090 10.1126/science.1127344

[b3] de Boer P., Hoogenboom J. P. & Giepmans B. N. G. Correlated light and electron microscopy: ultrastructure lights up! Nat. Methods 12, 503–513 (2015). 26020503 10.1038/nmeth.3400

[b4] Paez-Segala M. G. . Fixation-resistant photoactivatable fluorescent proteins for CLEM. Nat. Methods 12, 215–218, 4 p following 218 (2015). 25581799 10.1038/nmeth.3225 PMC4344411

[b5] Collman F. . Mapping synapses by conjugate light-electron array tomography. J. Neurosci. 35, 5792–5807 (2015). 25855189 10.1523/JNEUROSCI.4274-14.2015 PMC4388933

[b6] Peddie C. J., Liv N., Hoogenboom J. P. & Collinson L. M. Integrated light and scanning electron microscopy of GFP-expressing cells. Methods Cell Biol. 124, 363–389 (2014). 25287850 10.1016/B978-0-12-801075-4.00017-3

[b7] Johnson E. . Correlative in-resin super-resolution and electron microscopy using standard fluorescent proteins. Sci. Rep. 5, 9583 (2015). 25823571 10.1038/srep09583 PMC4379466

[b8] Watanabe S. . Protein localization in electron micrographs using fluorescence nanoscopy. Nat. Methods 8, 80–84 (2011). 21102453 10.1038/nmeth.1537 PMC3059187

[b9] Tokuyasu K. T. Immunochemistry on ultrathin frozen sections. Histochem. J. 12, 381–403 (1980). 7440248 10.1007/BF01011956

[b10] Kopek B. G., Shtengel G., Grimm J. B., Clayton D. A. & Hess H. F. Correlative photoactivated localization and scanning electron microscopy. PLoS One 8, e77209 (2013). 24204771 10.1371/journal.pone.0077209 PMC3808397

[b11] Vicidomini G. . A novel approach for correlative light electron microscopy analysis. Microsc. Res. Tech. 73, 215–224 (2010). 19725102 10.1002/jemt.20777

[b12] Loussert Fonta C. . Analysis of acute brain slices by electron microscopy: a correlative light-electron microscopy workflow based on Tokuyasu cryo-sectioning. J. Struct. Biol. 189, 53–61 (2015). 25448886 10.1016/j.jsb.2014.10.011

[b13] Bos E. . Vitrification of Tokuyasu-style immuno-labelled sections for correlative cryo light microscopy and cryo electron tomography. J. Struct. Biol. 186, 273–282 (2014). 24704216 10.1016/j.jsb.2014.03.021

[b14] Takizawa T. & Robinson J. M. Correlative microscopy of ultrathin cryosections in placental research. Methods Mol. Med. 121, 351–369 (2006). 16251754 10.1385/1-59259-983-4:349

[b15] Cortese K., Diaspro A. & Tacchetti C. Advanced correlative light/electron microscopy: current methods and new developments using Tokuyasu cryosections. J. Histochem. Cytochem. 57, 1103–1112 (2009). 19654103 10.1369/jhc.2009.954214 PMC2778083

[b16] Griffiths G., McDowall A., Back R. & Dubochet J. On the preparation of cryosections for immunocytochemistry. J. Ultrastruct. Res. 89, 65–78 (1984). 6544882 10.1016/s0022-5320(84)80024-6

[b17] Karreman M. A., Van Donselaar E. G., Agronskaia A. V., Verrips C. T. & Gerritsen H. C. Novel contrasting and labeling procedures for correlative microscopy of thawed cryosections. J. Histochem. Cytochem. 61, 236–247 (2013). 23264637 10.1369/0022155412473756 PMC3636698

[b18] Heuser J. Three-dimensional visualization of coated vesicle formation in fibroblasts. J. Cell Biol. 84, 560–583 (1980). 6987244 10.1083/jcb.84.3.560 PMC2110580

[b19] Suleiman H. . Nanoscale protein architecture of the kidney glomerular basement membrane. Elife 2, e01149 (2013). 24137544 10.7554/eLife.01149 PMC3790497

[b20] Bos E. . A new approach to improve the quality of ultrathin cryo-sections; its use for immunogold EM and correlative electron cryo-tomography. J. Struct. Biol. 175, 62–72 (2011). 21473917 10.1016/j.jsb.2011.03.022

[b21] Horstmann H., Körber C., Sätzler K., Aydin D. & Kuner T. Serial section scanning electron microscopy (S3EM) on silicon wafers for ultra-structural volume imaging of cells and tissues. PLoS One 7, e35172 (2012). 22523574 10.1371/journal.pone.0035172 PMC3327660

[b22] Wurm C. A. . Nanoscale distribution of mitochondrial import receptor Tom20 is adjusted to cellular conditions and exhibits an inner-cellular gradient. Proc. Natl. Acad. Sci. USA 108, 13546–13551 (2011). 21799113 10.1073/pnas.1107553108 PMC3158204

[b23] Schmidt R. . Mitochondrial cristae revealed with focused light. Nano Lett. 9, 2508–2510 (2009). 19459703 10.1021/nl901398t

[b24] Sorensen M. V. . Rapid dephosphorylation of the renal sodium chloride cotransporter in response to oral potassium intake in mice. Kidney Int. 83, 811–824 (2013). 23447069 10.1038/ki.2013.14

[b25] Loussert Fonta C. & Humbel B. M. Correlative microscopy. Arch. Biochem. Biophys. 581, 98–110 (2015). 26072116 10.1016/j.abb.2015.05.017

[b26] Collman F. . Mapping synapses by conjugate light-electron array tomography. J. Neurosci. 35, 5792–5807 (2015). 25855189 10.1523/JNEUROSCI.4274-14.2015 PMC4388933

[b27] Slot J. W. & Geuze H. J. Cryosectioning and immunolabeling. Nat. Protoc. 2, 2480–2491 (2007). 17947990 10.1038/nprot.2007.365

[b28] Causton B. E. Inthe science of biological specimen preparation for microscopy and microanalysis (ed. Müller M., Becker R.P., Boyde A.) 209–214 (Scanning electron microscopy, 1985).

[b29] Walther P. & Müller M. Biological ultrastructure as revealed by high resolution cryo-SEM of block faces after cryo-sectioning. J. Microsc. 196, 279–287 (1999). 10594768 10.1046/j.1365-2818.1999.00595.x

[b30] Goldstein J. I. Scanning electron microscopy and X-ray microanalysis: a text for biologists, materials scientists, and geologists. (Plenum Press, 1992).

[b31] Schwarz H. & Gorb S. Method of platinum-carbon coating of ultrathin sections for transmission and scanning electron microscopy: an application for study of biological composites. Microsc. Res. Tech. 62, 218–224 (2003). 14506687 10.1002/jemt.10343

[b32] Hell S., Reiner G., Cremer C. & Stelzer E. H. K. Aberrations in confocal fluorescence microscopy induced by mismatches in refractive index. J. Microsc. 169, 391–405 (1993).

[b33] Diaspro A., Annunziata S. & Robello M. Single-pinhole confocal imaging of sub-resolution sparse objects using experimental point spread function and image restoration. Microsc. Res. Tech. 51, 464–468 (2000). 11074617 10.1002/1097-0029(20001201)51:5<464::AID-JEMT9>3.0.CO;2-D

[b34] Kopek B. G., Shtengel G., Xu C. S., Clayton D. A. & Hess H. F. Correlative 3D superresolution fluorescence and electron microscopy reveal the relationship of mitochondrial nucleoids to membranes. Proc. Natl. Acad. Sci. USA 109, 6136–6141 (2012). 22474357 10.1073/pnas.1121558109 PMC3341004

[b35] Vancová M. & Nebesářová J. Correlative Fluorescence and Scanning Electron Microscopy of Labelled Core Fucosylated Glycans Using Cryosections Mounted on Carbon-Patterned Glass Slides. PLoS One 10, e0145034 (2015). 26690057 10.1371/journal.pone.0145034 PMC4699470

[b36] Cardona A. . TrakEM2 software for neural circuit reconstruction. PLoS One 7, e38011 (2012). 22723842 10.1371/journal.pone.0038011 PMC3378562

[b37] Sochacki K. A., Shtengel G., van Engelenburg S. B., Hess H. F. & Taraska J. W. Correlative super-resolution fluorescence and metal-replica transmission electron microscopy. Nat. Methods 11, 305–308 (2014). 24464288 10.1038/nmeth.2816 PMC3943662

[b38] Schindelin J. . Fiji: an open-source platform for biological-image analysis. Nat. Methods 9, 676–682 (2012). 22743772 10.1038/nmeth.2019 PMC3855844

