# Supplementary material for: Topographic contrast of ultrathin cryo-sections for correlative super-resolution light and electron microscopy
Source: Sci Rep. 2016 Sep 26;6:34062. doi: 10.1038/srep34062 (PMC5036093; doi:10.1038/srep34062)
Supplement: Supplementary Information [file srep34062-s1.pdf]

Topographic contrast of ultrathin cryo-sections for correlative super-resolution light and electron microscopy

José María Mateos, Bruno Guhl, Jana Doehner, Gery Barmettler, Andres Kaech, Urs Ziegler

Supplementary figures

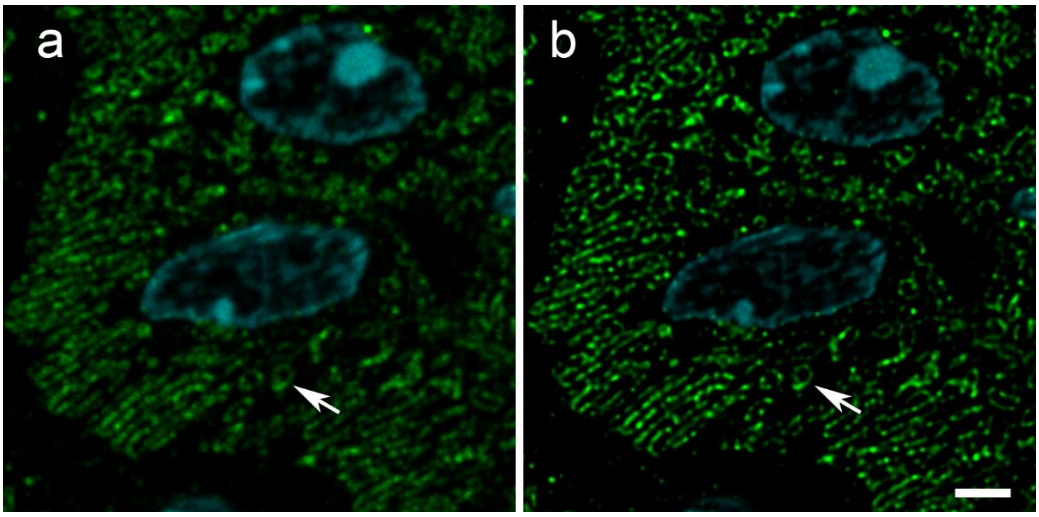

Supplementary Figure 1. Comparison between confocal microscopy and deconvolved images. **a**. Mitochondrial staining (Tom20; green) on a mouse kidney cryo-section and DAPI imaged with a confocal microscope. **b**. Same image after deconvolution (Huygens). Image appears less blurred and with a better signal to noise ratio. Arrows points to cross section of a mitochondrion in both images. Scale: 2 μm.

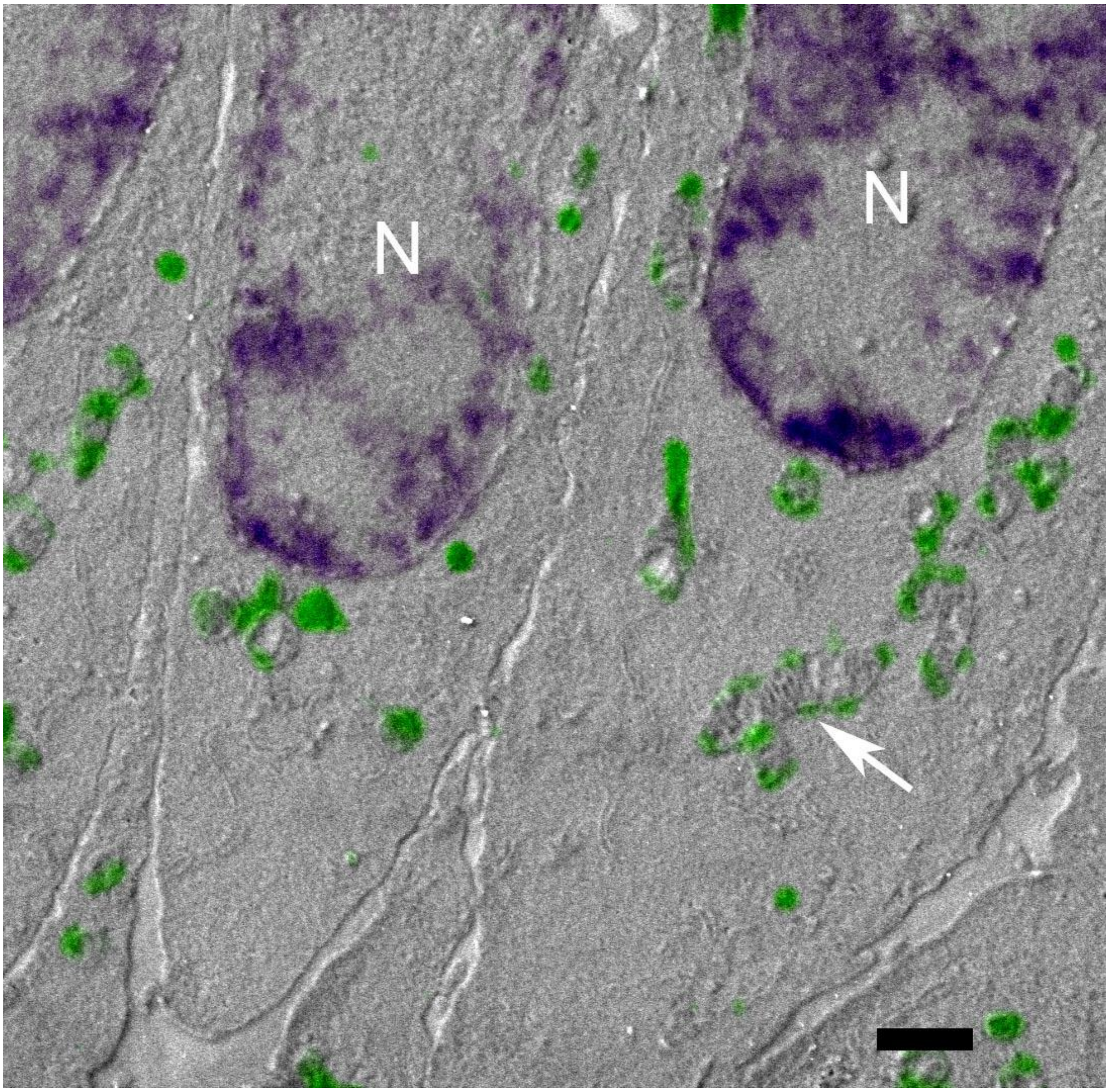

Supplementary Figure 2. CLEM on HeLa cells showing Tom20 expression (green) on the outer membrane of mitochondria (confocal microscopy followed by deconvolution) and the correlation with the mitochondria on the SEM image. Arrow points to a mitochondrion labelled with Tom20 and mitochondrial cristae are detected by SEM imaging. DAPI signal (purple) correlates with the nuclei (N) on the SEM image. Scale: 1 μm.

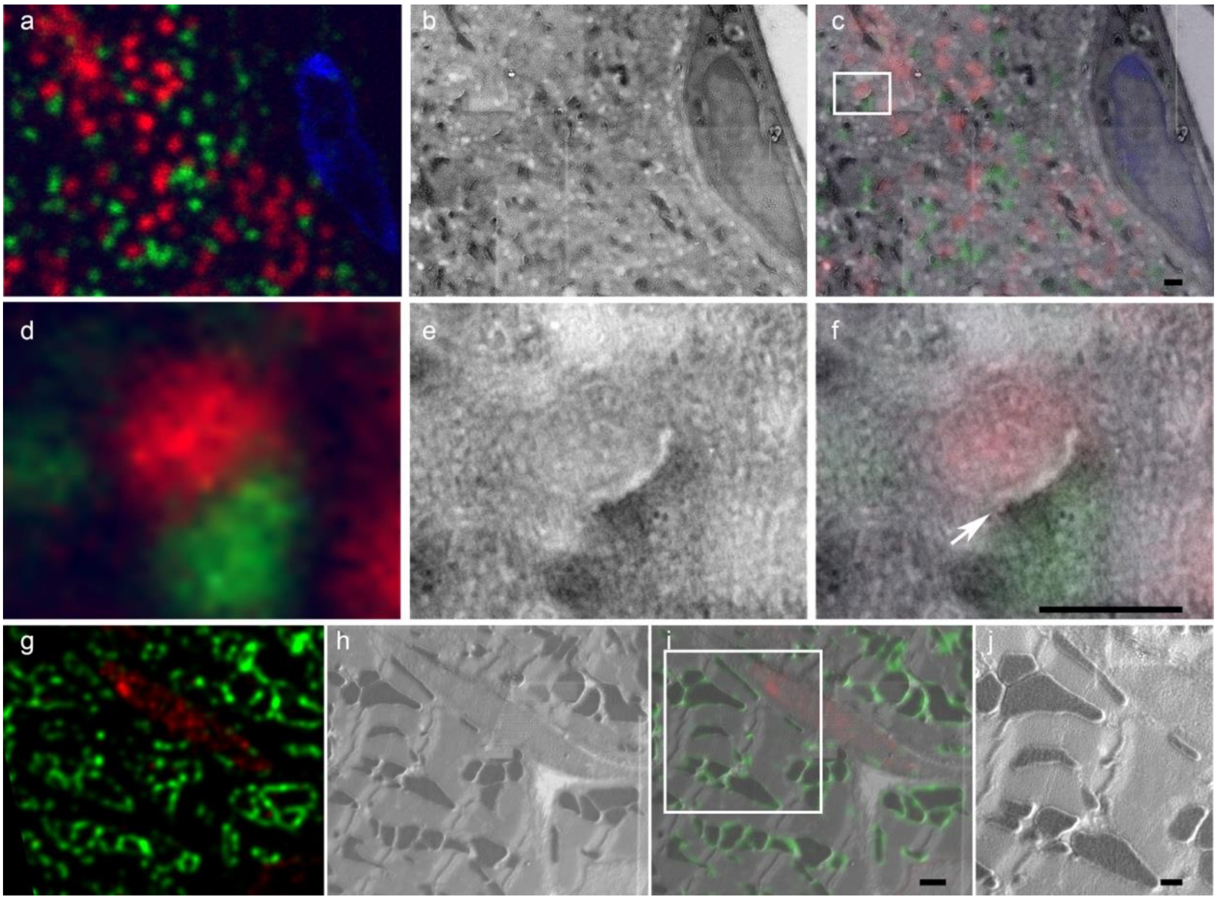

Supplementary Figure 3. CLEM on mouse cerebellar and heart Tokuyasu sections. **a**. Pre- and post-synaptic elements were labelled with V-GluT1 (green) and Calbindin (red) respectively, imaged with a confocal microscope and then deconvolved. DAPI (blue) labels nucleus. **b**. SEM image of the same section. **c**. Merge registered image of a and b. **d**. Detail of white box in c with double immunofluorescence showing pre- and post-synaptic staining. **e**. SEM image from white box in c. **f**. Merged image correlates the synaptic cleft detected by SEM with the closely detected pre- and post-synaptic signals (arrow). **g**. Heart Tokuyasu section with mitochondria stained with Tom20 (green) and nucleus with DAPI (red) imaged with a confocal microscope and deconvolved. **h**. SEM image of the same section. **i**. Merge registered image of g and h. **j** Detail of white box in i showing mitochondria and sarcomere. Scale bars: c; f and j 0.5 μm; i 1 μm.

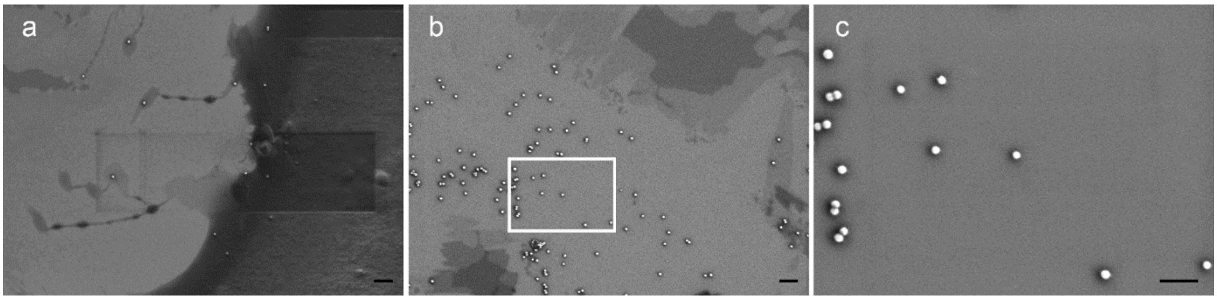

Supplementary Figure 4. Conditions for methylcellulose application. Methylcellulose was applied at 4°C during 1-2 min on wafers containing tetraspeck beads (100nm) and centrifuged at 2 conditions. **a**. After centrifugation at 1000x g for 30 sec. methylcellulose was

irregularly dispersed and flat areas appeared close to large thick methylcellulose surfaces. On these areas (right side of image) imaging was difficult and some of the beads were occluded by the methylcellulose. **b**. At 14100g for 90 sec (conditions used for all experiments) the surface was homogeneous **c**. High magnification of white box in b showing the beads. Scale bars: a and b: 1 μm; c: 0.5 μm.

Supplementary material and methods

Supplementary Figure 2. HeLa cells were cultivated in Petri dishes and fixed with 2% formaldehyde and 0.025% glutaraldehyde in cacodylate buffer (0.1 M, pH 7.4) overnight, incubated in 0.5% BSA, scraped off and centrifuged at 500 x g for 2 min. The pellet was re-suspended in 12% gelatine in 0.1 M PB at 42 °C and centrifuged at 800 x g for 3 min followed by polymerizing the gelatine for 30 min on ice water. 1 mm^3 pieces were cut on ice-cold sucrose solution and infiltrated in 2.3 M sucrose in PB (0.02 M) for 16 hours at 4 °C. The rest of the steps are the same as in Material and Methods.
